# Relation of four nontraditional lipid profiles to diabetes in rural Chinese H-type hypertension population

**DOI:** 10.1186/s12944-017-0590-7

**Published:** 2017-10-11

**Authors:** Haoyu Wang, Xiaofan Guo, Yintao Chen, Zhao Li, Jiaqi Xu, Yingxian Sun

**Affiliations:** Department of Cardiology, The First Hospital of China Medical University, 155 Nanjing North Street, Heping District, Shenyang, 110001 Liaoning People’s Republic of China

**Keywords:** H-type hypertension, Lipid ratios, Diabetes, Lipids, Rural population

## Abstract

**Background:**

Mounting evidence suggested that nontraditional lipid profiles have been recognized as a reliable indicator for unfavorable cardiovascular events. The purpose of this study was to explore the role of nontraditional lipid profiles as potential clinical indices for the assessment of prevalent diabetes in rural Chinese H-type hypertension population.

**Methods:**

During 2012 to 2013, we conducted a large cross-sectional study of 2944 H-type hypertension participants (≥35 years of age) from rural areas in northeast China. Subjects underwent accurate assessment of lipid profiles, fasting plasma glucose (FPG), homocysteine (Hcy) according to standard protocols.

**Results:**

The proportion of diabetes showed a graded and linear increase across the quartiles for all four nontraditional lipid parameters. Nontraditional lipid variables were independent determinants of FPG, and its correlation for TG/HDL-C was strongest, whether potential confounders were adjusted or not. Multivariable logistic regression analysis established that the highest triglycerides (TG)/ high-density lipoprotein cholesterol (HDL-C) quartile manifested the largest ORs of prevalent diabetes (OR: 3.275, 95%CI: 2.109–5.087) compared with the lowest quartile. The fully adjusted ORs (95%CI) were 2.753 (1.783–4.252), 2.178 (1.415–2.351), 1.648 (1.097–2.478) for the top quartile of total cholesterol (TC)/HDL-C, low-density lipoprotein cholesterol (LDL-C)/HDL-C, and non-high-density lipoprotein cholesterol (non-HDL-C), respectively. On the basis of the area under receiver-operating characteristic curve (AUC), TG/HDL-C showed the optimal discriminating power for diabetes (AUC: 0.684, 95% CI: 0.650–0.718).

**Conclusions:**

Nontraditional lipid profiles (TG/HDL-C, TC/HDL-C, LDL-C/HDL-C and non-HDL-C) were all consistently and independently correlated with prevalent diabetes among the H-type hypertension population in rural China. TG/HDL-C was prone to be more profitable in assessing the risk of prevalent diabetes and should be encouraged as an effective clinical tool for monitoring and targeted intervention of diabetes in H-type hypertension adults.

## Background

Hypertension is recognized as one of the major causes of cardiovascular disease (CVD) and mortality in worldwide [1]. In China, it has been regarded as the second most common leading risk factor of disability-adjusted life-years and deaths [2]. H-type hypertension is the concept of the concurrence of hypertension and high homocysteine (HHcy) by its concentration ≥ 10 μmol/L [3–5]. A multi-community, randomized study from the China Stroke Primary Prevention Trial revealed that H-type hypertension accounted for 80.3% of the hypertensive patients in 20,702 adults [6]. Previous studies suggested that H-type hypertension has been proposed to be an independent risk factor for carotid atherosclerotic plaques and cardio-cerebrovascular events [3, 4, 7–9]. The dramatically increasing prevalence of H-type hypertension is a great challenge to public health concerns and constitutes a serious socioeconomic burden. It was well-known that diabetes, being equivalent to the risk of coronary heart disease (CHD), is a major contributor to CVD and stroke [10, 11]. Clinical observations suggested that hypertensive subjects with elevated Hcy levels were positively linked with insulin resistance and the development of diabetes [12–15]. The association of H-type hypertension with diabetes might be relevant for a synergistic risk for vascular diseases. Hence, identifying risk factors with regard to diabetes in H-type hypertension population could help improve population-based strategies for screening and prevention of subjects who are predisposed to be at increased risk of vascular complications.

Traditional lipid parameters, represented by decreased high-density lipoprotein cholesterol (HDL-C) and hypertriglyceridemia, has been confirmed to be a common finding in individuals with diabetes [16–18]. Studies have proved that better control of dyslipidemia show favorable effect on vascular disease and related cardiovascular mortality in the subjects with diabetes [19, 20]. It has recently been proposed that nontraditional lipid profiles are powerful predictor for cardio-cerebrovascular diseases [21–25]. For instance, TG/HDL-C represents a highly atherogenic marker of insulin resistance and cardiometabolic risk, which correlates positively with LDL phenotype B and LDL particle concentration, and inversely with small dense LDL particle size [24, 26]. The role of triglyceride (TG)/HDL-C in relation to cardiovascular disorders and mortality has been well documented [21, 22, 27, 28]. Moreover, total cholesterol (TC)/HDL-C, low-density lipoprotein cholesterol (LDL-C)/HDL-C, and non-HDL-C have found to be independent indicator of vascular risk with greater predictive value than isolated lipid levels [23–25, 29]. Nevertheless, the association of nontraditional lipid parameters with diabetes remains unsettled. To date, the positive relationship of TG/HDL-C with diabetes has been indentified in a few prospective studies [30–33], whereas one survey reported that the influence of TG/HDL on diabetes occurrence has been poor [34]. Apart from this, epidemiological evidence revealed that participants with elevated TC/HDL-C and non-HDL-C levels had a more unfavorable chance of developing diabetes [35, 36]. However, nontraditional lipid profiles and its association with diabetes in H-type hypertension population have not been investigated.

In this scenario, to highlight the clinical significance of nontraditional lipid indices (TG/HDL-C, TC/HDL-C, LDL-C/HDL-C, non-HDL-C) in developing stages of cardio-cerebrovascular events, our current study aims to evaluate whether nontraditional lipid profiles were capable of identifying diabetes among hypertensive adults with HHcy levels in rural China. We also compared the attributes of four nontraditional lipid variables and attempted to determine the extent to which parameter is an optimal surrogate for identifying diabetic subjects who are more likely to be at increased risk of cardio-cerebrovascular disease.

## Methods

### Study population

The data originated from a large population-based epidemiological cross-sectional study of 11,956 permanent residents (age ≥ 35) in rural areas of China. The full details regarding the study design and definitions were extensively described elsewhere [37–39]. This study enrolled 2944 H-type hypertension participants with complete data on nontraditional lipid variables and diabetes. The Ethics Committee of China Medical University (Shenyang, China) approved the study protocol. Each participant was contacted and informed in advance to know the purpose and procedure of survey before obtaining written informed consent.

### Data collection and measurements

The detailed process about data collection and methods selection of this sample has been completely illuminated in our prior publications [37–39]. All subjects fulfilled a structured questionnaire regarding on demographic characteristics, socioeconomic data, lifestyle risk factors, medical history of stroke and CVD, and medication used in the past two weeks with the assistance of cardiologists and trained nurses.

Based on the recommended AHA’s protocol, after each subject had been resting at least 5 min, blood pressure (BP) were measured by trained observer in a sitting position. The three consecutive measurements were collected on the right arm at 2-min intervals and the average value was recorded for analysis.

Anthropometric assessments were acquired including items of weight, height, and waist circumference (WC). Height and weight were accurate to 0.1 cm and 0.1 kg, respectively with the individuals wearing light clothing and bare feet. WC was measured by placing an inelastic tape metre in umbilical level at minimal respiration. Body mass index (BMI) was calculated as weight/height^2^ (kg/m^2^).

After an overnight fasting with 12 h, the Blood sample was drawn from each participant antecubital vein in the morning for assessing fasting plasma glucose (FPG), serum creatinine (SCr), TC, TG, LDL-C, and HDL-C. Details of Storage process and laboratory measurement methods were available in the previous publications [37–39]. Plasma Hcy was measured by enzymatic cycling assay (Hitachi, Japan). Non-HDL-C levels were determined by subtracting serum HDL-C levels from TC [30, 35]. TG/HDL-C, TC/HDL-C, LDL-C/HDL-C were measured as TG, TC, LDL-C divided by HDL-C.

### Definitions

According to the JNC-7 report [40], diagnosis of hypertension was established as an average systolic blood pressure (SBP) at least 140 mmHg or/and diastolic blood pressure (DBP) ≥90 mmHg or individuals who were on antihypertensive medications or self-reported previous diagnosed hypertension. The definition of diabetes was determined as follows: fasting glucose greater than or equal to 7.0 mmol/L or self-reported medical diagnosis history or receiving plasma glucose lowering therapy in the light of American Diabetes Association criteria [41]. HHcy was identified by the concentration of Hcy ≥10 mmol/L [42, 43]. Individuals with concomitant hypertension and HHcy were defined as having H-type hypertension [3, 5].

### Statistical analyses

The H-type hypertension population is expressed as the means with standard deviations (SDs) or numbers with percentages. Comparisons between individuals stratified by diabetes were assessed with Student’s t-test to examine differences in means, while Chi-squared test for independence was utilized to compare differences of categorical variables in proportions. Each of non-traditional lipid profile was stratified into quartiles in accordance with the distribution of lipid variables. The chi-square linear-by-linear association test was analyzed to test for linear trends across the quartile groups of nontraditional lipid parameters for proportions of diabetes. Pearson’s correlation analysis regarding the association of two variables was performed. The relationship of nontraditional lipid parameters with FPG levels as a continuous variable was evaluated by stepwise multivariate regression analysis, which is expressed through standardized regression coefficient. We conducted the logistic regression analyses to evaluate the odds ratios (ORs) and corresponding 95% confidence intervals (CIs) of prevalent diabetes in H-type hypertension population by the quartiles of TG/HDL-C, TC/HDL-C, LDL-C/HDL-C, and non-HDL-C with adjustment for various confounding risk factors. The receiver-operating characteristic (ROC) curve was employed to determine the predictive value of each nontraditional four lipid profiles on diabetes. Assessing the discrimination ability of nontraditional lipid variables in detecting diabetes was performed by the area under the ROC curve (AUC). All of the statistical analyses involved the application of SPSS software, version 22.0 (IBM Corp), and a two-tailed *P* < 0.05 was adopted to be statistically significant.

## Results

Clinical and demographic characteristics of H-type hypertension population recruited in this survey, as stratified by diabetes status, are presented in Table 1. A total of 2944 individuals (1960 males and 984 females) were included in the present study, with 272 cases (9.2%) of diabetes identified. The average age of participants with diabetes was 59.84 years (SD = 9.85), 3 year older than the rest of the participants. When compared to subjects without prevalent diabetes, the diabetes group were more likely to be low physical activity, higher BMI, and higher WC (*P* < 0.05). The mean values of SBP and DBP were remarkably elevated in those with diabetes than those without (*P* < 0.001). The participants with diabetes exhibited significantly higher levels of TG/HDL-C, TC/HDL-C, LDL-C/HDL-C, and non-HDL-C (P < 0.001). Additionally, participants with diabetes had a higher prevalence of the history of stroke, cardiovascular disease and medication used.

**Table 1:** Characteristics of H-type hypertension population with or without diabetes

Variables	Total (*n* = 2944)	Non-diabetes (*n* = 2672)	Diabetes (*n* = 272)	*P* value
Age (years)	57.09 ± 11.29	56.81 ± 11.39	59.84 ± 9.85	<0.001
Male (%)	1960 (66.6)	1789 (67.0)	171 (62.9)	0.174
Race				0.065
Han (%)	2751 (93.4)	2504 (93.7)	247 (90.8)	
Others (%)	193 (6.6)	168 (6.3)	25 (9.2)	
Education				0.231
low	1529 (51.9)	1375 (51.5)	154 (56.6)	
middle	1147 (39.0)	1049 (39.3)	98 (36.0)	
high	268 (9.1)	248 (9.3)	20 (7.4)	
Family income (CNY/year)				0.340
≤ 5000	652 (22.1)	590 (22.1)	62 (22.8)	
5000–20,000	1648 (56.0)	1506 (56.4)	142 (52.2)	
> 20,000	644 (21.9)	576 (21.6)	68 (25.0)	
Physical activity				0.021
low	2559 (86.9)	2337 (87.5)	222 (81.6)	
moderate	290 (9.9)	251 (9.4)	39 (14.3)	
high	95 (3.2)	84 (3.1)	11 (4.0)	
Current smoking status (%)	1429 (48.5)	1306 (48.9)	123 (45.2)	0.250
Current drinking status (%)	882 (30.0)	793 (29.7)	89 (32.7)	0.297
Diet score	2.26 ± 1.17	2.27 ± 1.17	2.20 ± 1.17	0.392
BMI (kg/m2)	24.75 ± 3.71	24.60 ± 3.70	26.21 ± 3.52	<0.001
WC (cm)	84.24 ± 9.88	83.74 ± 9.83	89.17 ± 9.01	<0.001
SBP (mmHg)	147.09 ± 25.58	145.88 ± 25.19	158.94 ± 26.37	<0.001
DBP (mmHg)	83.37 ± 12.46	83.08 ± 12.34	86.26 ± 13.30	<0.001
SCr (mmol/L)	75.74 ± 28.34	75.60 ± 218.88	77.70 ± 22.33	0.407
FPG (mmol/L)	5.82 ± 1.56	5.46 ± 0.57	9.29 ± 3.17	<0.001
TG (mmol/L)	1.38 ± 1.59	1.28 ± 1.33	2.35 ± 2.99	<0.001
TC (mmol/L)	3.79 ± 1.14	3.72 ± 1.06	4.40 ± 1.62	<0.001
LDL-C (mmol/L)	2.14 ± 0.76	2.11 ± 0.73	2.45 ± 0.90	<0.001
HDL-C (mmol/L)	1.43 ± 0.44	1.45 ± 0.44	1.30 ± 0.42	<0.001
TG/HDL-C ratio (mmol/L)	1.38 ± 1.59	1.28 ± 1.33	2.35 ± 2.99	<0.001
TC/HDL-C ratio (mmol/L)	3.79 ± 1.14	3.72 ± 1.06	4.40 ± 1.62	<0.001
LDL-C/HDL-C ratio (mmol/L)	2.14 ± 0.76	2.11 ± 0.73	2.45 ± 0.90	<0.001
non-HDL-C (mmol/L)	3.66 ± 0.99	3.62 ± 0.97	4.04 ± 1.10	<0.001
History of stroke (%)	350 (12.2)	289 (11.1)	61 (22.8)	<0.001
History of CVD (%)	451 (15.7)	384 (14.7)	67 (25.1)	<0.001
Medication used ^a^ (%)	1658 (56.3)	1479 (55.4)	179 (65.8)	0.001

Data are expressed as the mean (SD) or as n (%). CNY, China Yuan (1CNY = 0.154 USD).

*BMI* body mass index, *WC* waist circumference, *SBP* systolic blood pressure, *DBP* diastolic blood pressure, *FPG* fasting plasma glucose, *TC* total cholesterol, *TG* triglyceride, *HDL-C* high-density lipoprotein cholesterol, *LDL-C* low-density lipoprotein cholesterol, *non-HDL-C* non-high-density lipoprotein cholesterol, *SCr* serum creatinine, *CVD* cardiovascular disease

^a^Indicating any self-reported medication used in the past two weeks

**Fig. 1:**
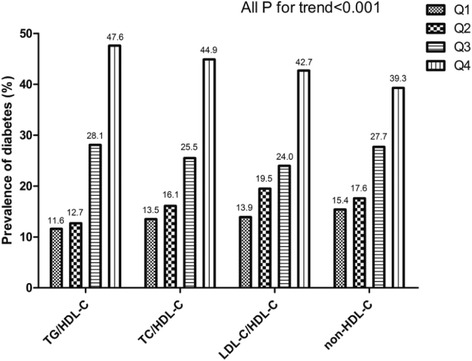
Prevalence of diabetes in H-type hypertension population by quartiles of TG/HDL-C, TC/HDL-C, LDL-C/HDL-C and non-HDL-C. A linear increasing trend across nontraditional lipid profiles quartile groups was observed

Figure 1 summarized the prevalence of diabetes in hypertensive participants with HHcy according to quartiles of nontraditional lipid parameters. Across the increasing quartiles of all nontraditional lipid variables, we found a significantly escalating linear trend in the proportion of diabetes (all P for trend <0.001). The prevalence of diabetes in H-type hypertension population increased 4.0-fold, 3.3-fold, 3.1-fold, 2.6-fold higher in the highest TG/HDL-C, TC/HDL-C, LDL-C/HDL-C and non-HDL-C group than in the lowest groups respectively.

As shown in Table 2, univariate correlation analysis revealed that TG/HDL-C, TC/HDL-C, LDL-C/HDL-C and non-HDL-C manifested significant positive correlations with FPG (all *P* < 0.001). FPG was also associated with age, physical activity, BMI, WC, SDP, DBP, history of stroke and CVD (all *P* < 0.05). By performing stepwise multivariate regression analysis, we found that all nontraditional lipid variables were still independent determinants of FPG in H-type hypertension population even after further adjustment for a broad array of potential confounders (all *P* < 0.001). The high values for standardized regression coefficient are indicative of strong correlations, which meant TG/HDL-C was the strongest variable that interacted independently on FPG.

**Table 2:** Pearson’s correlation and stepwise multivariate regression analysis of nontraditional lipid profiles and fasting plasma glucose in subjects with H-type hypertension

Variables	FPG (mmol/L)									
			Model 1		Model 2		Model 3		Model 4	
	r	*P* value	β	*P* value	β	*P* value	β	*P* value	β	*P* value
Age	0.104	<0.001	0.073	<0.001	0.054	0.006	0.049	0.017	0.049	0.014
Sex	−0.007	NS	NI		NI		NI		NI	
Race	0.014	NS	NI		NI		NI		NI	
Education	−0.028	NS	NI		NI		NI		NI	
Family income	−0.013	NS	NI		NI		NI		NI	
Physical activity	0.048	0.009	–	NS	–	NS	–	NS	–	NS
Current smoking status	−0.026	NS	NI		NI		NI		NI	
Current drinking status	0.035	NS	NI		NI		NI		NI	
Diet score	−0.025	NS	NI		NI		NI		NI	
BMI	0.097	<0.001	–	NS	–	NS	−0.061	0.042	–	NS
WC	0.152	<0.001	0.094	<0.001	0.083	<0.001	0.142	<0.001	0.104	<0.001
SBP	0.152	<0.001	0.097	<0.001	0.108	<0.001	0.104	<0.001	0.095	<0.001
DBP	0.108	<0.001	–	NS	–	NS	–	NS	–	NS
Scr	0.022	NS	NI		NI		NI		NI	
TG	0.187	<0.001	NI		NI		NI		NI	
TC	0.117	<0.001	NI		NI		NI		NI	
LDL-C	0.091	<0.001	NI		NI		NI		NI	
HDL-C	−0.086	<0.001	NI		NI		NI		NI	
History of stroke	0.086	<0.001	–	NS	–	NS	–	NS	–	NS
History of CVD	0.068	<0.001	0.042	0.021	0.037	0.047	0.043	0.021	0.043	0.021
Medication used	0.033	NS	NI		NI		NI		NI	
TG/HDL-C ratio	0.169	<0.001	0.142	<0.001	NI		NI		NI	
TC/HDL-C ratio	0.173	<0.001	NI		0.129	<0.001	NI		NI	
LDL-C/HDL-C ratio	0.148	<0.001	NI		NI		0.098	<0.001	NI	
non-HDL-C	0.158	<0.001	NI		NI		NI		0.104	<0.001
R^2^			0.064		0.060		0.054		0.055	

Variables were included in multivariate models when their *p* value was less than 0.05 in Pearson’s correlation; Model 1: age, physical activity, BMI, WC, SBP, history of stroke and CVD, TG/HDL-C ratio were considered as independent variables; Model 2: age, physical activity, BMI, WC, SBP, history of stroke and CVD, TC/HDL-C ratio were considered as independent variables; Model 3: age, physical activity, BMI, WC, SBP, history of stroke and CVD, LDL-C/HDL-C ratio were considered as independent variables; Model 4: age, physical activity, BMI, WC, SBP, history of stroke and CVD, non-HDL-C were considered as independent variables

*Abbreviations: r* correlation coefficient, *β* regression coefficient, *BMI* body mass index, *WC* waist circumference, *SBP* systolic blood pressure, *DBP* diastolic blood pressure, *SCr* serum creatinine, *TC* total cholesterol, *TG* triglyceride, *HDL-C* high-density lipoprotein cholesterol, *LDL-C* low-density lipoprotein cholesterol, *HDL-C* high-density lipoprotein cholesterol, *non-HDL-C* non-high-density lipoprotein cholesterol, *CVD* cardiovascular disease, *NS* not significant, *NI*, not included in the model

Table 3 revealed that the ORs and 95%CI for the presence of diabetes all showed a significant progressive increase across higher TG/HDL-C, TC/HDL-C, LDL-C/HDL-C and non-HDL-C levels in a dose-response fashion (all P for trend < 0.05). After multivariable adjustment, H-type hypertension subjects in the top quartile of TG/HDL-C had 3.3-fold increased odds of prevalent diabetes relative to those in the bottom quartile of TG/HDL-C (OR: 3.275, 95%CI: 2.109–5.087), while the smallest ORs for highest non-HDL-C quartile with regard to diabetes was 1.648 (95%CI: 1.097–2.478). Meanwhile, the ORs of prevalent diabetes increased by a factor of 2.8 for H-type hypertension individuals in the highest quartile of TC/HDL-C group (OR: 2.753, 95%CI: 1.783–4.252) as compared with the reference group. The association strength for prevalent diabetes in hypertensive adults with HHcy was slightly lower in the highest quartile of LDL-C/HDL-C group (OR: 2.178, 95%CI: 1.415–3.351).

**Table 3:** Odd ratios (95% CI) for diabetes in hypertensive participants with elevated homocysteine levels according to quartiles of nontraditional lipid profiles

Variables	Lipid measures				P for trend
	Quartile 1	Quartile 2	Quartile 3	Quartile 4	
TG/HDL-C ratio	≤0.59	0.59–0.92	0.92–1.55	>1.55	
Model 1	1.000 (reference)	1.094 (0.670–1.787)	2.552 (1.667–3.906)	4.604 (3.079–6.885)	<0.001
Model 2	1.000 (reference)	1.020 (0.623–1.670)	2.420 (1.578–3.711)	4.574 (3.054–6.852)	<0.001
Model 3	1.000 (reference)	0.955 (0.573–1.590)	2.044 (1.299–3.125)	3.275 (2.109–5.087)	<0.001
TC/HDL-C ratio	≤3.12	3.12–3.80	3.80–4.58	>4.58	
Model 1	1.000 (reference)	1.189 (0.757–1.868)	1.980 (1.310–2.993)	3.841 (2.619–5.634)	<0.001
Model 2	1.000 (reference)	1.161 (0.738–1.826)	1.882 (1.243–2.850)	3.673 (2.501–5.396)	<0.001
Model 3	1.000 (reference)	1.123 (0.702–1.797)	1.682 (1.079–2.621)	2.753 (1.783–4.252)	<0.001
LDL-C/HDL-C ratio	≤1.64	1.64–2.11	2.11–2.65	>2.65	
Model 1	1.000 (reference)	1.457 (0.948–2.239)	1.729 (1.141–2.618)	3.520 (2.403–5.156)	<0.001
Model 2	1.000 (reference)	1.424 (0.925–2.190)	1.648 (1.087–2.500)	3.363 (2.292–4.933)	<0.001
Model 3	1.000 (reference)	1.207 (0.769–1.894)	1.361 (0.874–2.120)	2.178 (1.415–3.351)	<0.001
non-HDL-C	≤3.11	3.11–3.71	3.71–4.43	>4.43	
Model 1	1.000 (reference)	1.203 (0.786–1.841)	1.830 (1.235–2.712)	2.832 (1.951–4.112)	<0.001
Model 2	1.000 (reference)	1.143 (0.745–1.751)	1.723 (1.160–2.559)	2.571 (1.761–3.755)	<0.001
Model 3	1.000 (reference)	0.902 (0.578–1.410)	1.315 (0.867–1.994)	1.648 (1.097–2.478)	0.002

Model 1: unadjusted; Model 2: adjusted for age and sex; Model 3: adjusted for all the factors in Model 2 and race, education level, family income, current smoking and drinking status, physical activity, diet score, body mass index, waist circumference, systolic blood pressure, diastolic blood pressure, serum creatinine, history of stroke and cardiovascular disease (coronary heart disease, arrhythmia and heart failure), medication used

*Abbreviations: OR* odd ratio, *95% CI* 95% confidence interval, *TC* total cholesterol, *TG* triglyceride, *HDL-C* high-density lipoprotein cholesterol, *LDL-C* low-density lipoprotein cholesterol, *non-HDL-C*, non-high-density lipoprotein cholesterol

Table 4 showed the AUCs (and 95% CIs) of diabetes in relation to the four nontraditional lipid profiles. TG/HDL-C with highest AUC scores presented the best accuracy in predicting diabetes (AUC: 0.684, 95% CI: 0.650–0.718, *P* < 0.001), whereas non-HDL-C showed the lowest AUC value for diabetes (AUC: 0.621, 95% CI: 0.585–0.657, P < 0.001). Despite no significant difference was observed while comparing the predictive capability of LDL-C/HDL-C to non-HDL-C, the accuracy of detecting diabetes in H-type hypertensive adults was statistically different between either two of nontraditional lipid variables after comparisons of AUC (all *P* < 0.05).

**Table 4:** The area under the curve (AUC) of nontraditional lipid profiles for the presence of diabetes in H-type hypertension population

Variables	AUC	(95%CI)	*P* value
TG/HDL-C ratio	0.684	(0.650–0.718)^a,b,c^	<0.001
TC/HDL-C ratio	0.659	(0.624–0.693)^b,c,d^	<0.001
LDL-C/HDL-C ratio	0.623	(0.587–0.659)^a,d^	<0.001
non-HDL-C	0.621	(0.585–0.657)^a,d^	<0.001

*Abbreviations: 95%CI* 95% confidence interval, *TC* total cholesterol, *TG* triglyceride, *HDL-C* high-density lipoprotein cholesterol, *LDL-C* low-density lipoprotein cholesterol, *non-HDL-C* non-high-density lipoprotein cholesterol

^a^Indicates a significant difference as compared to TC/HDL-C

^b^Indicates a significant difference as compared to LDL-C/HDL-C

^c^Indicates a significant difference as compared to non-HDL-C

^d^Indicates a significant difference as compared to TG/HDL-C

## Discussion

In this large sample of community members, our main finding was that all nontraditional lipid profiles were significantly related to an increased risk for diabetes among rural H-type hypertension population in northeast China. FPG appeared to be correlated with nontraditional lipid profiles even after adjustment for the effect of covariates in multiple regression analysis. Positive and linear trend associations across increasing quartiles of nontraditional lipid parameters with the prevalence of diabetes were observed. Our study for the first time claimed that TG/HDL-C was thought to have a comparatively superior predictive ability in the identification of prevalent diabetes compared to TC/HDL-C, LDL-C/HDL-C, and non-HDL-C. To a greater importance, it was clear that detection of nontraditional lipid profiles might result beneficial for better prevention and control of diabetes in a large group of H-type hypertension adults.

Epidemiologic studies have demonstrated that lipoprotein abnormalities correlated independently with the presence of diabetes [16, 18, 44]. Lipid-lowering therapy has largely contributed to a better reduction of major vascular events and cardiovascular risk in patients with diabetes [20, 45]. Recently, nontraditional lipid profiles have become available to predict cardio-cerebrovascular disease for making clinical decisions in prospective studies, which communicate the higher risk for cardiometabolic disease progression at an early stage [21–24, 27, 29]. Arsenault BJ and colleagues supported that independently of LDL-C, subjects with increased TC/HDL-C and non-HDL-C levels conferred an increased CHD risk [23]. Another study conducted in Asia proved the potential use of TG/HDL-C and TC/HDL-C for CVD risk prediction [22]. Moreover, LDL-C/HDL-C has been considered as a valuable and standard indicator for CVD with greater predictive effect than each parameter used independently [25, 46]. For instance, TC/HDL-C has been suggested as an indirect estimate of atherogenic lipoprotein particle concentration and size, which was not accessible in simple lipid variables [23, 29]. At present, the findings of a large cross-sectional study consisting of representative US civilian population sample described that combination of elevated Hcy and hypertension delivered a 12.0-fold and 17.3-fold higher risk of stroke in males and females, respectively [9]. It was worth noting that elevated homocysteine levels combined with hypertension had a substantially higher risk of developing early carotid artery atherosclerosis and cardio-cerebrovascular events [3, 7–9], as well as exerted a jointed effect with diabetes on the risk of stroke and CVD [12–15]. Thus, it is conceivable that the H-type hypertensive subjects with diabetes are easily subjected to future risk of cardiovascular diseases and mortality. However, the role of nontraditional lipid variables with respect to the presence of diabetes among the hypertensive population with hyperhomocysteinemia has not been elucidated. Consequently, this large, contemporary population-based survey is initiated to analyze the influence of nontraditional lipid profiles on diabetes in large-scale H-type hypertension population of China.

A study based on city community found an independent contribution of per 1 SD increment in TG/HDL-C to prevalent diabetes (OR: 1.45, 95%CI: 1.31–1.60) [36]. Hadaegh et al. also found that there was positive association of TG/HDL-C with prevalent diabetes among Iranian population, and those with 1 SD change in TG/HDL-C were 1.4 times and 1.3 times more likely to have prevalent diabetes in females and males, respectively [30]. Similarly, a cohort study of Japanese community residents in that the risks of diabetes were significantly augmented with elevated TG/HDL-C levels in developed country [31]. In 687 participants from an urban community in China, TG/HDL-C had a significant difference in the risk prediction of diabetes occurrence with the ORs being 1.341 (*p* = 0.010) [47]. In accordance with their results, our study reported that participants with the highest TG/HDL-C levels conferred 3.3-fold greater odds of prevalent diabetes than those with the lowest quartile in H-type hypertension population. However, one prospective study showed a null association, which meant TG/HDL-C failed to predict the incidence of diabetes among high-risk individuals in Iran [34]. Inconsistent evidence from previous studies can be explained by the fact that the survey was performed in Iranian people who experienced a greater risk of diabetes occurrence, resulting from having first degree relatives of patients with diabetes.

The data from a Canada residents showed that there were pronounced effects of increased TC/HDL-C and non-HDL-C levels on odds of prevalent diabetes, and non-HDL-C was suggested to be the most useful marker of prevalent diabetes among other lipid profiles [35]. Consistently, our study confirmed that the independent and positive effect of TC/HDL-C and non-HDL-C on likelihood of diabetes in H-type hypertension adults, in which the accuracy of TC/HDL-C identifying diabetes was superior over non-HDL-C. Additional, there has been no relevant literature confirming a significant predictive value of LDL-C/HDL-C in relation to diabetes in hypertensive subjects with elevated homocysteine levels so far. Our study provided a novel insight that LDL-C/HDL-C provided a positive correlation with diabetes among H-type hypertension populations in China.

The mechanism by which the association between nontraditional lipid profiles and diabetes might be related arouses great interest. It was widely-accepted that lipotoxicity, inflammation, and endoplasmic reticulum (ER) stress can induce insulin resistance (IR) [48–50]. Previous studies have revealed that high TG levels seem to give rise to a remarkable amount of fatty acids to be deposited in cells in ectopic lipid storage [51]. Unger RH, et al. indicated that hypertriglyceridemia has a highly significant association with lipotoxicity, which cause overload of free fatty acid (FFA) levels in the skeletal muscle and pancreatic islets, as well as lead to β-cell dysfunction, apoptosis, insulin resistance and diabetes [52]. Lipotoxicity is responsible for the main causal mechanism of IR [48, 49, 53]. Besides, dyslipidemia could directly promote inflammation or ER stress that serves as a possible causal factor of IR [16]. Moreover, low HDL-C levels may have a negative impact on glucose homeostasis through reducing insulin secretion, weakening insulin sensitivity, and impairing the process of glucose uptake by muscle via the AMP-activated protein kinase [53]. It has been shown that elevated LDL-C levels promote the loss of β-cells in spite of being impossible to modulate insulin sensitivity [54, 55]. IR is also a common pathway related to the formation of increased VLDL, as a component of non-HDL-C, and plasma FFA levels, which stimulates lipogenesis and cholesterol synthesis. Notably, the excess amounts of FFA are generated in adipose tissue and transferred to the liver, which brings about the raise production of VLDL and TG, increased clearance of HDL-C by kidney, and a decline of HDL-C levels under the influence of cholesterol ester transfer protein [56, 57]. Accordingly, it is obvious that dyslipidemia and IR form a vicious, reciprocal feedback cycle, and they may reinforce each other. This interaction accelerates the development of IR in the early stages of the development of diabetes.

Some limitations and strengths of the present study need to be mentioned when interpreting our results. Firstly, the cross-sectional design limits our capacity to make causal inference between nontraditional lipid parameters and diabetes in H-type hypertension population, so future longitudinal studies to investigate the potential role of CMI as a predictor are warranted. Secondly, we failed to collect information on vitamin B12, folic acid, and methylenetetrahydrofolate reductase (MTHFR) genotype, which would be possible confounders affecting H-type hypertension population. Furthermore, data on using fasting plasma glucose as the criterion for judging for the presence of diabetes has been extensively established in the large-scale epidemiological survey [58, 59], but our present study did not measure random blood glucose or hemoglobin A1c, which might reduce the accuracy of diagnosis of diabetes. Likewise, the absence of fasting insulin, HOMA-IR, and HOMA-Beta may reduce the possibility of screening all patients with diabetes. However, it is noteworthy that our population-based design for the first time confirms that nontraditional lipid profiles (TG/HDL-C, TC/HDL-C, LDL-C/HDL-C, and non-HDL-C) make a valuable contribution to a much greater risk for diabetes in H-type hypertension population of rural China. These data are particularly meaningful in the case of hypertensive Chinese rural population with high homocysteine levels who are more prone to an increased risk of atherosclerosis and cardiovascular ischemic events. The advantages of using nontraditional lipid variables measurements are inexpensive and are easy to calculate in annual health examination.

## Conclusions

In summary, our study revealed in H-type hypertension population of rural Northeast China that TG/HDL-C, TC/HDL-C, LDL-C/HDL-C, and non-HDL-C were all positively and significantly associated with the risk of prevalent diabetes independent of relevant confounding factors. Also, TG/HDL-C appeared to outperform other nontraditional lipid parameters in determining the presence of diabetes. These results underscored the necessity of adopting nontraditional lipid parameters as a convenient indicator for clinical settings of diabetes in Chinese adults with H-type hypertension.

## Abbreviations

AUC: Area under receiver-operating characteristic curve
BMI: Body mass index
CHD: Coronary heart disease
CVD: Cardiovascular disease
DBP: Diastolic blood pressure
ER: Endoplasmic reticulum
FPG: Fasting plasma glucose
Hcy: Homocysteine
HDL-C: High-density lipoprotein cholesterol
IR: Insulin resistance
LDL-C: Low-density lipoprotein cholesterol
non-HDL-C: Non-high-density lipoprotein cholesterol
OR: Odd ratio
SBP: Systolic blood pressure
SCr: Serum creatinine
SD: Standard deviation
TC: Total cholesterol
TG: Triglyceride
WC: Waist circumference
